# The Longevity Improvement & Fair Evidence (LIFE) Study: Overview of the Study Design and Baseline Participant Profile

**DOI:** 10.2188/jea.JE20210513

**Published:** 2023-08-05

**Authors:** Haruhisa Fukuda, Chieko Ishiguro, Rei Ono, Kosuke Kiyohara

**Affiliations:** 1 Department of Health Care Administration and Management, Kyushu University Graduate School of Medical Sciences, Fukuoka, Japan; 2 Center for Cohort Studies, Kyushu University Graduate School of Medical Sciences, Fukuoka, Japan; 3 Section of Clinical Epidemiology, Department of Data Science, Center for Clinical Sciences, National Center for Global Health and Medicine, Tokyo, Japan; 4 Department of Public Health, Kobe University Graduate School of Health Sciences, Hyogo, Japan; 5 Department of Food Science, Otsuma Women’s University, Tokyo, Japan

**Keywords:** study profile, database project, real-world data, Japan, LIFE Study

## Abstract

**Background:**

The Longevity Improvement & Fair Evidence (LIFE) Study, which was launched in 2019, is a multi-region community-based database project that aims to generate evidence toward extending healthy life expectancy and reducing health disparities in Japan. Herein, we describe the LIFE Study’s design and baseline participant profile.

**Methods:**

Municipalities participating in the LIFE Study provide data from government-administered health insurance enrollees and public assistance recipients. These participants cover all disease types and age groups. Centered on healthcare claims data, the project also collects long-term care claims data, health checkup data, vaccination records, residence-related information, and income-related information. The different data types are converted into a common data model containing five modules (health care, long-term care, health checkup, socioeconomic status, and health services). We calculated the descriptive statistics of participants at baseline in 2018.

**Results:**

The LIFE Study currently stores data from 1,420,437 residents of 18 municipalities. The health care module contains 1,280,756 participants (mean age: 65.2 years), the long-term care module contains 189,069 participants (mean age: 84.3 years), and the health checkup module contains 274,375 participants (mean age: 69.0 years). Although coverage and follow-up rates were lower among younger persons, the health care module includes 74,151 children (0–19 years), 273,157 working-age adults (20–59 years), and 933,448 older persons (≥60 years).

**Conclusion:**

The LIFE Study provides data from over 1 million participants and can facilitate a wide variety of life-course research and cohort studies. This project is expected to be a useful platform for generating real-world evidence from Japan.

## INTRODUCTION

With one of the highest life expectancies and lowest infant mortality rates, Japan consistently ranks among the world’s best performers in key health indicators.^1^ Furthermore, the Japanese government continues to work toward building a vibrant society in which people lead healthy and enriched lives. In 2013, the government implemented the “Health Japan 21 (The Second Term)” plan, which features a 10-year roadmap to promote health within the Japanese population in the 21st century.^2^ This plan also specifies two major national health policy targets: the extension of healthy life expectancy and the reduction of health disparities.

In 2013, the Cabinet of Japan adopted the “Japan Revitalization Strategy”, which introduced a system that requires all insurers to develop, disclose, implement, and evaluate health plans based on analyses of administrative claims data and health checkup data.^3^ Under the National Health Insurance system (which covers one in every four Japanese people), municipal governments fulfill the roles of insurers and act as command centers to coordinate and support problem-solving efforts for enrollees’ health-related issues. Moreover, these municipal governments have a broad and in-depth cognizance of their residents’ general health statuses through routine screenings and health services, such as prenatal checkups, infant health checkups, vaccinations, specific health checkups (ie, annual checkups for persons aged 40–74 years to identify those at risk of lifestyle diseases), cancer screening, and dental checkups. Based on this information, municipal public health nurses are able to work toward preventing the onset of disease (primary prevention) and progression to symptomatic disease (secondary prevention) in at-risk residents. Municipal governments must also cover an increasing number of long-term care (LTC) insurance enrollees due to population aging. Although the LTC insurance system for persons aged ≥75 years is operated by transregional insurance associations organized at the prefectural level, the 2020 amendment to the Health Insurance Act introduced the integrated implementation of health services and LTC prevention services for older persons.^4^ Under this scheme, municipal governments are also tasked with conducting health promotion services for older residents. As a result of these legal and data-related frameworks, municipal governments are ideally positioned to play a leading role in solving health-related problems in Japan.

However, the municipal governments are currently unable to fully utilize the health care insurance data, LTC insurance data, health data, and government administration data that they routinely collect and store. This could be due to a variety of reasons, such as the lack of resident-specific IDs shared across data types; the heterogeneity of the various data formats, which preclude consolidated analyses; and the separation of municipal agencies according to life stage without any inter-agency department that can coordinate studies. In order to overcome these barriers and facilitate analyses of the available data, we launched the Longevity Improvement & Fair Evidence (LIFE) Study in 2019 as a project to measure, evaluate, and propose solutions for health-related problems within community residents in Japan using a life-course perspective. As the LIFE Study is intended to be used by a large number of researchers in the future, this paper describes its design, data items, and participant profile at baseline.

## METHODS

### Rationale and study design

The primary aim of the LIFE Study is to contribute to Japan’s health policy targets of extending healthy life expectancy and reducing health disparities. To achieve this aim, the LIFE Study seeks to facilitate research at the municipal level, which represents the front line of providing healthcare to the community. Accordingly, the LIFE Study also possesses characteristics of a social project that supports health services, health care, and LTC care in municipalities using an integrated data science approach. Research using the LIFE Study can include analyses on disease prevalences and expenditures according to region, disease type, or patient characteristics, as well as analyses on the effectiveness or economic viability of various preventive care and health care programs. These analytical results are then presented as feedback to the participating municipalities.

The LIFE Study also aims to promote database-derived epidemiological studies and life-course epidemiological studies through collaborative projects with other research institutions. After a municipality enters into an agreement with the LIFE Study, it provides health-related data from National Health Insurance enrollees, Latter-Stage Older Persons Health Care System enrollees, LTC Insurance enrollees, and public assistance recipients. Individuals can be followed-up through these data unless they move out of the municipality, change insurers, or die. The LIFE Study continuously collects, processes, and stores these data in a database. The database is then used to provide datasets to researchers for various studies with the overarching aim of generating evidence that can help to extend healthy life expectancy and reduce health disparities.

**Figure 1.**
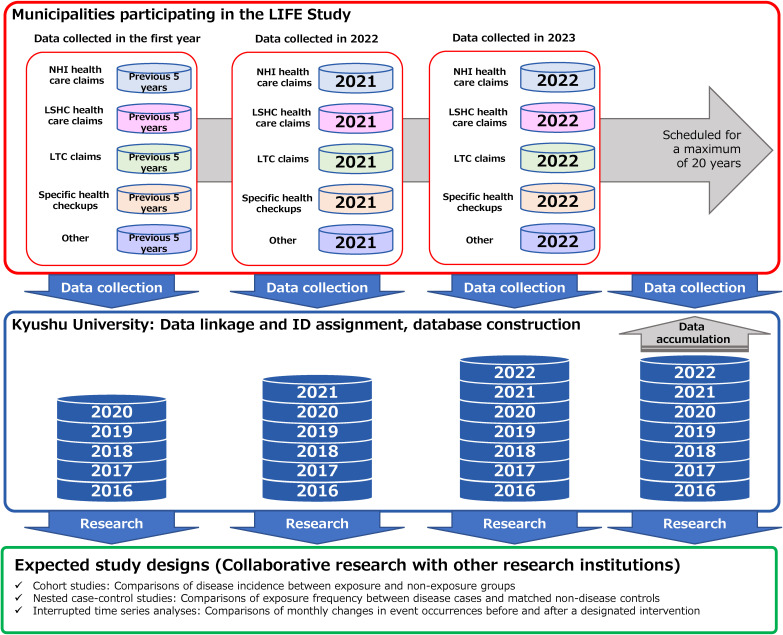
Overview of the LIFE Study design. LSHC, Latter-Stage Older Persons Health Care System; LTC, long-term care; NHI, National Health Insurance.

Figure 1 shows an overview of the LIFE Study design, including data collection, database construction, and research applications. In the first year of entering the LIFE Study, a municipality will prepare and provide health-related data from the past 5 years to Kyushu University. In each subsequent year, the municipalities provide data from the previous year. The data are merged at the resident level and stored in a database at Kyushu University. Researchers at Kyushu University and collaborating research institutions use the data to conduct various types of studies, such as cohort studies, nested case-control studies, and interrupted time-series analyses.

The LIFE Study was approved by the Kyushu University Institutional Review Board for Clinical Research (Observational Research: 2021-399). Approval for data use was obtained from each municipality’s Personal Information Protection Review Board.

### Development of the LIFE Study database

The LIFE Study is a multi-region community-based database project conducted by Kyushu University (Fukuoka, Japan), and is scheduled to run for 20 years beginning from 2019. The project is conducted under individual agreements between Kyushu University and each participating municipality. The LIFE Study collects National Health Insurance claims data, Latter-Stage Older Persons Health Care System claims data, LTC Insurance claims data, and specific health checkups and specific health guidance data from the municipalities. Each municipal government requests for and acquires these data in the pre-anonymized format from the National Health Insurance Federation that oversees their prefecture. Public assistance data and other government administration data are obtained directly from each municipality’s information systems. LIFE Study staff visit the municipalities to collect and process health care insurance data, LTC insurance data, health data, and government administration data. First, the staff assign a unique research ID code (resident ID 1: RTID1) to each resident, and these codes are used to link individuals across the various data types. The assignment of RTID1 codes is performed within each municipal government’s building, with oversight by government staff. Next, personally identifiable information is removed before the data are sent to Kyushu University. Each municipal government creates a correspondence table that links the RTID1 codes with their matching residents’ names, but these tables are not shared at any time with LIFE Study researchers or other third parties. The correspondence tables are created only for the municipal governments to notify residents with health-related problems (identified in feedback from the analytical results) and to provide opt-out opportunities for those who do not wish to participate in the study. At Kyushu University, the RTID1 codes are further transformed into RTID2 codes, and only the latter are included in the analytical database for research purposes. There are no correspondence tables for RTID2 codes and residents’ names, which ensures that researchers cannot link the RTID2 codes in the LIFE Study data with the corresponding residents’ names stored by the municipal governments.

The participants and data items to be collected are specified in the agreement with each municipal government. Therefore, the LIFE Study only receives and stores data that these governments agree to share. The LIFE Study compiles the collected data into datasets, which are provided to research groups for analysis. Before these datasets are delivered, the institution(s) of each research group must enter into data utilization agreements and submit data operational regulations to Kyushu University. There are also measures to ensure that the research groups use the data in accordance with these agreements and regulations.

### Participants

As of December 2021, 18 municipalities are participating in the LIFE Study. These include 15 municipalities from Fukuoka Prefecture, one municipality from Oita Prefecture, one municipality from Hyogo Prefecture, and one municipality from Tokyo Metropolis. The number of participating municipalities is projected to increase in the future.

The LIFE Study’s target participants include National Health Insurance enrollees, Latter-Stage Older Persons Health Care System enrollees, LTC Insurance enrollees, and public assistance recipients. These participants cover the entire range of age groups from birth to death. However, each participating municipality individually determines the types of participants to be included in the LIFE Study. For example, some municipalities only choose to provide data on participants aged ≥40 years, whereas others do not include (or only include) public assistance recipients.

Data collection from the municipalities began in April 2014. However, the National Health Insurance Federation, which governs matters related to the secondary use of insurance-related data, stipulates a 5-year storage limitation for claims data. Therefore, each municipality’s starting date of data availability is dependent on its date of participation in the LIFE Study.

### Data collection

**Figure 2.**
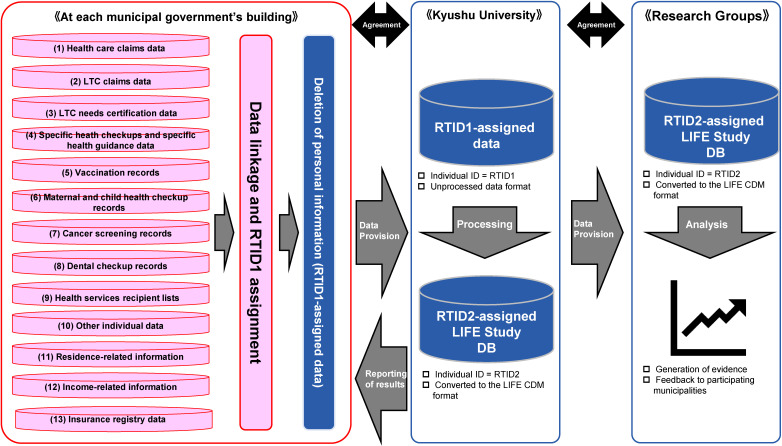
Flowchart of data collection in the LIFE Study. CDM, common data model; DB, database, LTC, long-term care.

Figure 2 shows a flowchart of data collection in the LIFE Study. The following data items are collected: (1) health care claims data, (2) LTC claims data, (3) LTC needs certification data, (4) specific health checkups and specific health guidance data, (5) vaccination records, (6) maternal and child health checkup records, (7) cancer screening records, (8) dental checkup records, (9) health services/LTC prevention services recipient lists, (10) other health care/LTC/health-related individual data, (11) residence-related information (elementary school district, junior high school district, and area of daily activities), (12) income-related information (out-of-pocket payment ceiling amount category under the High-Cost Medical Expense Benefit System and insurance premium category for primary insured persons), and (13) insurance registry data. However, there are inter-municipal variations in the governmental departments assigned to participate in the LIFE Study, resulting in differences in the data items provided by each municipality.

In each municipal government’s building, we generate an RTID1 code for each resident using the following four items from the Basic Resident Register: name in kanji (ie, Japanese ideographic characters), name in kana (ie, Japanese syllabic characters), sex, and date of birth. In each data type, individuals that correspond to these four items are assigned the same RTID1 code. Subsequently, all personally identifiable information is deleted from the data types, which are then sent to Kyushu University. RTID1 codes are not assigned to individuals if (1) there are multiple residents with the same kanji names, kana names, sex, and date of birth, or (2) there are no matching residents in the data types.

At Kyushu University, the collected data are converted into a LIFE Common Data Model (CDM) to facilitate data integration and provision to researchers for analyses. Here, RTID1 codes are transformed into RTID2 codes, and datasets are generated for research. As shown in Table 1, the LIFE CDM is composed of five modules: a health care module, an LTC module, a health checkup module, a socioeconomic status module, and a health services module. The health care module contains 16 tables, the LTC module contains 17 tables, the health checkup module contains six tables, the socioeconomic status module contains three tables, and the health services module contains two tables. To facilitate a better understanding of the LIFE CDM, we briefly explain the main tables in the health care module. In the KAN table, one record exists for each RTID2 code, which enables researchers to ascertain the number of unique individuals with health care claims data at any point in time. Next, the REC table contains claims-specific records, which researchers can use to acquire information on medical expenditures, numbers of days of care, occurrence of death, and insurance type for each health care encounter. In the SYO table, one record exists for each diagnosis. In addition to all recorded diagnoses, the table also contains labels to identify suspected diagnoses and main diagnoses. As the health care claims data only include Japanese diagnosis codes, Kyushu University adds the corresponding International Classification of Diseases, 10th Revision (ICD-10) codes using a master file for medical fee payments provided by the Ministry of Health, Labour and Welfare.^8^ The “*utagai byoumei*” and “*syubyoumei*” labels from the health care claims data are copied to the SYO table and used to identify suspected diagnoses and main diagnoses, respectively. The SIN table contains one record per treatment, and researchers can identify the dates and number of times each treatment was received by a patient. In the IYA table, one record exists for each prescribed drug. The information in this table includes drug codes, prescription dates, and dosages for all prescribed drugs. As the health care claims data only include Japanese drug codes, Kyushu University adds the corresponding therapeutic classes (first three digits of the National Health Insurance Drug Price Standard codes) using a master file for medical fee payments provided by the Ministry of Health, Labour and Welfare.^8^ In each table, the original data files are checked for the numbers of records according to month and year to identify any missing data.

**Table 1.** Overview of the LIFE Common Data Model

Module	Table	Main types of information
Health care	KAN	Patient age, sex, dates of initial claims records, dates of final claims records
	REC	Medical expenditures, number of days of care, death, insurance types
	SYO	Diagnoses, suspected diagnoses, main diagnoses
	SIN	Types of received treatments, treatment dates, number of times treatments were received
	IYA	Prescribed drugs, prescription dates, dosages
	TOK	Special treatment materials used, use dates, quantities
	TYO	Types of dispensing procedures, use dates, quantities
	TKA	Additional fees for dispensing procedures, use dates, reimbursement points
	BUD	Diagnosis Procedure Combination codes, admission dates, discharge dates, outcomes
	IRK	Medical institution names, medical institution addresses
	ADM	Admission dates, discharge dates, hospitalization expenditures, in-hospital death
	COM	Charlson Comorbidity Index,^5^^,^^6^ Elixhauser Comorbidity Index^6^^,^^7^
	SSN	Types of received dental treatments, treatment dates, number of times treatments were received
	SSK	Dental formula codes
	SRK	Clinical departments
	KOH	Public expenses recipient registration numbers, amount of public expenses used
LTC	H1	Age, sex, LTC needs levels, LTC expenditures
	D1	[LTC services] Types of LTC services, number of units used, number of days/times services were used
	D2	[Short-term admission for recuperation in LTC health facilities] Dates of treatment initiation, number of days of doctor’s visits, number of days admitted, reimbursement points for rehabilitation services, reimbursement points for procedures, reimbursement points for surgery, etc
	D3	[Medical LTC sanatoria/LTC health facilities] Number of times insurance was used for specific treatment expenditures and special medical expenditures, number of units used, etc
	D4	[Facilities covered by LTC insurance] Meal expenditures
	D5	[In-home LTC support/LTC prevention support] Types of LTC services, number of units of LTC benefits used, designated care manager registration numbers
	D6	[Expenditures for welfare equipment covered by public expenses] Types of welfare equipment, dates of purchase, cost of purchases, product names
	D7	[Expenditures for home modification] Dates of home modification, names of contractors used, modification costs, etc
	D8	[Expenditures for high-cost LTC services] Amount borne by users, payment amounts, etc
	D9	[eg, facilities covered by LTC insurance] Service codes/number of units of LTC benefits used, payment ceiling amounts, billing claims for insurance, number of days, etc
	DA	Information related to expenditure reduction for social welfare corporations: service type codes, reduction rates, reduced amounts
	DB	[LTC prevention/comprehensive services for daily living support] Care management expenditures for LTC prevention
	DC	[LTC health facilities] Dates of emergency treatment initiation, number of days of doctor’s visits, number of days admitted, reimbursement points for rehabilitation services, reimbursement points for procedures, reimbursement points for surgery, etc
	DD	[LTC services for persons with LTC needs in which domicile exceptions apply] Types of LTC services, number of units used, number of days/times services were used
	T1	Tabulated information according to LTC service type
	NIN	Activities of daily living, LTC needs, period of certification
	NED	Status of social engagement
Health checkup	KEN	[Specific health checkups] Test items in the specific health checkup, questionnaire forms
	SID	[Specific health guidance] Content of specific health guidance
	HOG1	[Survey on households that receive public assistance] Minimum cost of living, amount of financial aid, reasons for starting/discontinuing public assistance
	HOG2	[Survey on household members that receive public assistance] Employment/school enrollment, medical aid
	BUB	[Maternal and child health] ^*^varies among the municipalities
	SCR	[Various health screenings] Screening types, screening dates, screening results (^*^varies among the municipalities)
Socioeconomic status	LOC	Elementary school districts, junior high school districts, and areas of daily activities
	INC_MED	Out-of-pocket payment ceiling amount categories under the High-Cost Medical Expense Benefit System (5 categories)
	INC_LTC	Insurance premium categories for primary insured persons (≥10 categories; varies among the municipalities)
Health services	VAC	Vaccination types, vaccination dates, institutions where vaccinations were administered, lot numbers
	YOB	Preventive care service names, dates

LTC, long-term care.

Almost all municipalities provide 5 years of data during the initial data collection. Over the targeted 20-year period of the LIFE Study, additional data from the previous year will be collected annually from the participating municipalities, and the datasets will be updated as appropriate.

### Statistical analysis

To provide an overview of the LIFE Study’s participant profile at baseline, we calculated the descriptive statistics using its database in fiscal year (FY) 2018. In Japan, the FY begins in April and ends in March of the following year. Therefore, FY2018 is from April 2018 through March 2019.

First, we calculated the distribution of participants within three selected tables from the modules according to age group (0–19, 20–39, 40–59, 60–79, and ≥80 years). Second, we used the diagnosis-related information from the SYO table in the health care module and calculated the distribution of diseases appearing in the LIFE Study according to ICD-10 codes and age group. For this analysis, we focused on confirmed diagnoses (including main diagnoses) and excluded all suspected diagnoses. There were participants with multiple diagnoses. Third, we calculated the distribution of participants according to certified LTC needs status (which include support needs levels 1–2 and LTC needs levels 1–5, with higher levels indicating greater care needs) and age group. As these levels may change over time in an individual participant, we only analyzed each participant’s highest level in FY2018. Consequently, these counts did not include participants who used LTC services without any certified LTC needs. Because LTC needs certification is given to persons aged ≤64 years with disabilities/debilitating diseases and to older persons aged ≥65 years, this factor was analyzed using the following age groups: ≤64, 65–74, 75–84, 85–94, and ≥95 years.

To assess the potential follow-up of the participants, we calculated each participant’s follow-up duration from the earliest recorded month until the latest recorded month within the health care claims data. Cases were censored if the latest recorded month was the same as the last month in the study period available in the database. Kaplan–Meier curves of the follow-up durations (months) were plotted according to age group in the earliest recorded month (0–19, 20–39, 40–59, 60–79, and ≥80 years).

## RESULTS

The LIFE Study currently receives and stores data from 18 municipalities. Although the different municipalities have different data collection start dates, the majority have provided 5 years of data beginning from either April 2014 or April 2015. From the health care claims data, we identified 1,420,437 persons with a unique RTID2 code that appeared at least once during the entire study period.

Table 2 shows the distribution of participants (identified through RTID2 codes in the data) according to age group in FY2018 for three selected tables within the LIFE CDM. In the REC table of the health care module, there were 1,280,756 participants from 18 municipalities in FY2018; their mean age was 65.2 years and women comprised 57.6% of the total. The highest proportion of participants were aged 60–79 years (47.7%), and 25.2% were aged ≥80 years. In the H1 table of the LTC module, there were 189,069 participants from 17 municipalities; their mean age was 84.3 years and women comprised 69.7% of the total. In the KEN table of the health checkup module, there were 274,375 participants with specific health checkup data from 17 municipalities; their mean age was 69.0 years and women comprised 59.0% of the total. All 720 participants in the 20–39 years group were aged 39 years; therefore, all participants in this group were actually 40 years of age during FY2018 (which spanned from April 2018 through March 2019).

**Table 2.** Distribution of LIFE Study participants according to age group in FY2018 for three selected tables in the LIFE Common Data Model

Module	Table	No. of municipalities	Overall	Age group (FY2018)				

				0–19 years	20–39 years	40–59 years	60–79 years	≥80 years
Health care	REC	18	1,280,756	74,151 (5.8%)	96,397 (7.5%)	176,760 (13.8%)	610,827 (47.7%)	322,621 (25.2%)
Long-term care	H1	17	189,069	0 (0%)	0 (0%)	1,354 (0.7%)	44,525 (23.5%)	143,190 (75.7%)
Health checkup	KEN	17	274,375	0 (0%)	720 (0.3%)	43,477 (15.8%)	197,420 (72.0%)	32,758 (11.9%)

FY, fiscal year.

The distribution of participants according to disease (ICD-10 codes) and age group is presented in Table 3. Proportions were calculated by setting the number of participants with each disease as the numerator and the total number of participants in the SYO table as the denominator. All diagnoses, with the exception of suspected diagnoses, were included. As a single participant can have multiple diagnoses, the proportions do not necessarily add up to 100%. The most common diagnoses were diseases of the digestive system (ICD-10 codes: K00–K93) with 1,058,971 participants, diseases of the respiratory system (J00–J99) with 765,776 participants, and diseases of the circulatory system (I00–I99) with 738,134 participants. Among the age groups, diseases of the respiratory system (ICD-10 codes: J00–J99) were the most common in younger participants aged 0–19 years, whereas diseases of the digestive system (K00–K93) were the most common in all other age groups.

**Table 3.** Distribution of LIFE Study participants according to disease and age group in FY2018

ICD-10 codes	Overall (*n* = 1,277,443)	Age group (FY2018)				

		0–19 y (*n* = 72,480)	20–39 y (*n* = 94,623)	40–59 y (*n* = 172,934)	60–79 y (*n* = 596,623)	≥80 y (*n* = 340,783)
A00–B99	415,824 (32.6%)	28,739 (39.7%)	23,518 (24.9%)	40,983 (23.7%)	186,896 (31.3%)	135,688 (39.8%)
C00–D48	251,719 (19.7%)	1,865 (2.6%)	6,131 (6.5%)	22,517 (13.0%)	132,932 (22.3%)	88,274 (25.9%)
D50–D89	184,179 (14.4%)	1,588 (2.2%)	5,340 (5.6%)	15,563 (9.0%)	77,292 (13.0%)	84,396 (24.8%)
E00–E90	735,693 (57.6%)	5,880 (8.1%)	15,767 (16.7%)	60,126 (34.8%)	391,575 (65.6%)	262,345 (77.0%)
F00–F99	288,947 (22.6%)	4,973 (6.9%)	14,599 (15.4%)	38,741 (22.4%)	116,146 (19.5%)	114,488 (33.6%)
G00–G99	516,280 (40.4%)	2,981 (4.1%)	14,438 (15.3%)	49,592 (28.7%)	237,502 (39.8%)	211,767 (62.1%)
H00–H59	558,931 (43.8%)	27,648 (38.1%)	24,787 (26.2%)	50,111 (29.0%)	275,885 (46.2%)	180,500 (53.0%)
H60–H95	174,574 (13.7%)	16,835 (23.2%)	6,556 (6.9%)	14,146 (8.2%)	79,674 (13.4%)	57,363 (16.8%)
I00–I99	738,134 (57.8%)	1,991 (2.7%)	5,074 (5.4%)	44,013 (25.5%)	390,240 (65.4%)	296,816 (87.1%)
J00–J99	765,776 (59.9%)	54,743 (75.5%)	48,849 (51.6%)	89,107 (51.5%)	345,985 (58.0%)	227,092 (66.6%)
K00–K93	1,058,971 (82.9%)	40,440 (55.8%)	57,735 (61.0%)	129,828 (75.1%)	517,667 (86.8%)	313,301 (91.9%)
L00–L93	556,749 (43.6%)	36,326 (50.1%)	30,963 (32.7%)	56,794 (32.8%)	243,428 (40.8%)	189,238 (55.5%)
M00–M99	720,739 (56.4%)	7,994 (11.0%)	17,442 (18.4%)	63,574 (36.8%)	362,258 (60.7%)	269,471 (79.1%)
N00–N99	387,057 (30.3%)	3,053 (4.2%)	17,749 (18.8%)	35,536 (20.5%)	176,036 (29.5%)	154,683 (45.4%)
O00–O99	5,088 (0.4%)	174 (0.2%)	3,920 (4.1%)	695 (0.4%)	185 (0.0%)	114 (0.0%)
P00–P96	2,008 (0.2%)	1,583 (2.2%)	324 (0.3%)	49 (0.0%)	23 (0.0%)	29 (0.0%)
Q00–Q99	36,277 (2.8%)	3,043 (4.2%)	1,561 (1.6%)	3,644 (2.1%)	16,893 (2.8%)	11,136 (3.3%)
R00–R99	587,348 (46.0%)	20,373 (28.1%)	23,253 (24.6%)	55,214 (31.9%)	274,597 (46.0%)	213,911 (62.8%)
S00–T98	551,424 (43.2%)	20,411 (28.2%)	14,896 (15.7%)	46,087 (26.7%)	268,461 (45.0%)	201,569 (59.1%)
V01–Y98	14 (0.0%)	0 (0.0%)	2 (0.0%)	6 (0.0%)	3 (0.0%)	3 (0.0%)
Z00–Z99	212,815 (16.7%)	238 (0.3%)	1,060 (1.1%)	5,045 (2.9%)	100,716 (16.9%)	105,756 (31.0%)

FY, fiscal year; ICD-10, International Classification of Diseases, 10th Revision.

Diseases were categorized according to ICD-10 codes: A00–B99, Certain infectious and parasitic diseases; C00–D48, Neoplasms; D50–D89, Diseases of the blood and blood-forming organs and certain disorders involving the immune mechanism; E00–E90, Endocrine, nutritional and metabolic diseases; F00–F99, Mental and behavioral disorders; G00–G99, Diseases of the nervous system; H00–H59, Diseases of the eye and adnexa; H60–H95, Diseases of the ear and mastoid process; I00–I99, Diseases of the circulatory system; J00–J99, Diseases of the respiratory system; K00–K93, Diseases of the digestive system; L00–L99, Diseases of the skin and subcutaneous tissue; M00–M99, Diseases of the musculoskeletal system and connective tissue; N00–N99, Diseases of the genitourinary system; O00–O99, Pregnancy, childbirth and the puerperium; P00–P96, Certain conditions originating in the perinatal period; Q00–Q99, Congenital malformations, deformations and chromosomal abnormalities; R00–R99, Symptoms, signs and abnormal clinical and laboratory findings, not elsewhere classified; S00–T98, Injury, poisoning and certain other consequences of external causes; V01–Y98, External causes of morbidity and mortality; Z00–Z99, Factors influencing health status and contact with health services.

Table 4 shows the distribution of participants according to certified LTC needs level and age group. In total, 185,430 participants with certified LTC needs were identified from the LTC claims dataset. Among these, 2,858 participants were aged ≤64 years. Overall, the most common LTC needs level was LTC needs level 1 with 32,540 participants. Among the age groups, the most common LTC needs levels were LTC needs level 2 in participants aged ≤64 years and 65–74 years, LTC needs level 1 in participants aged 75–84 years and 85–94 years, and LTC needs level 4 in participants aged ≥95 years.

**Table 4.** Distribution of LIFE Study participants according to certified LTC needs level and age group in FY2018

LTC needs level	Overall	Age group (FY2018)				

		≤64 years	65–74 years	75–84 years	85–94 years	≥95 years
Support needs level 1	21,521	201	2,373	9,543	8,919	485
Support needs level 2	27,400	476	3,297	10,766	11,928	933
LTC needs level 1	32,540	375	2,918	12,032	15,573	1,642
LTC needs level 2	31,537	584	3,281	10,483	14,724	2,465
LTC needs level 3	25,674	407	2,443	7,582	12,296	2,946
LTC needs level 4	26,287	393	2,189	7,348	12,630	3,727
LTC needs level 5	20,471	422	1,935	5,679	9,489	2,946
Overall	185,430	2,858	18,436	63,433	85,559	15,144

FY, fiscal year; LTC, long-term care.

**Figure 3.**
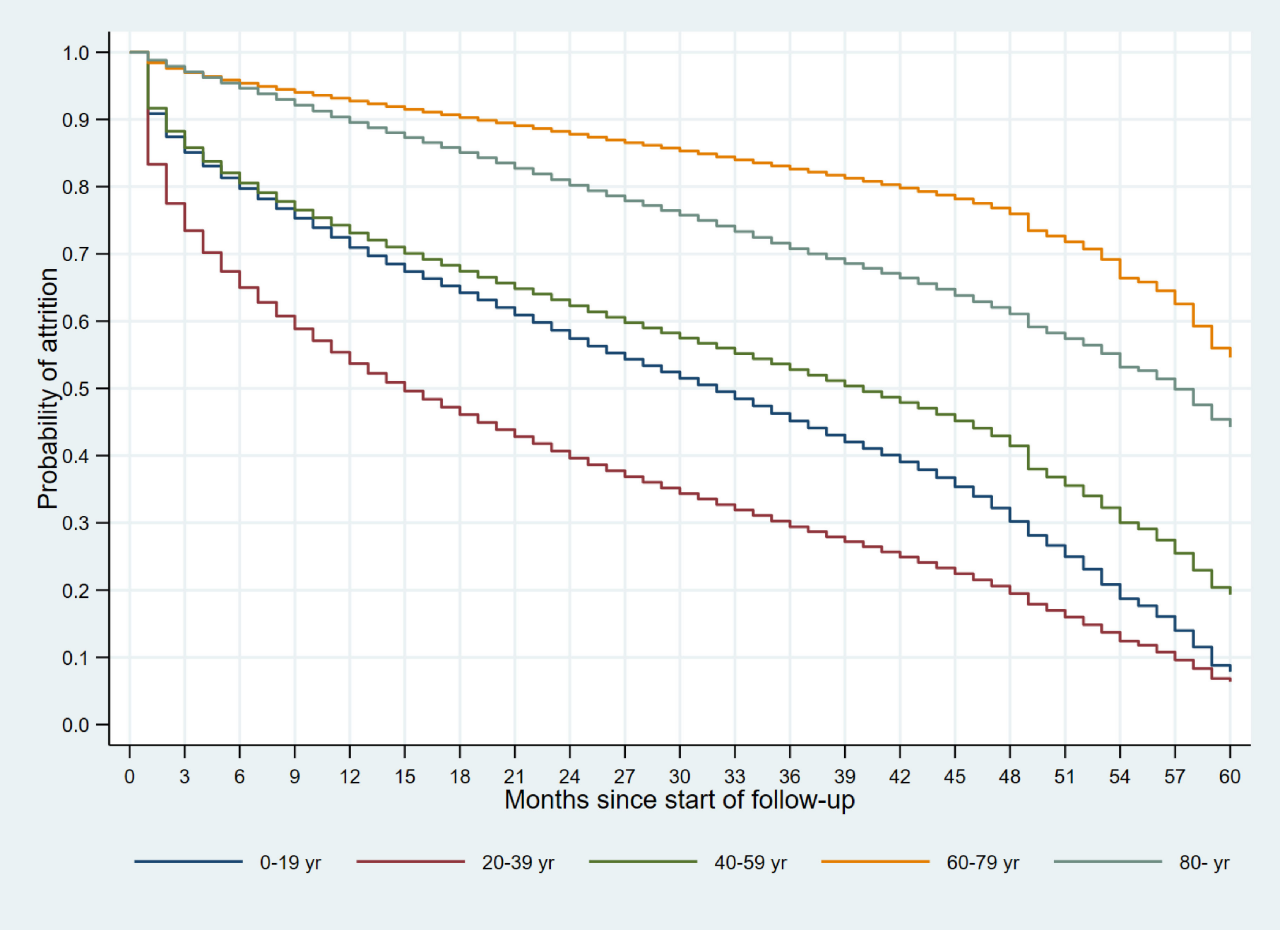
Kaplan–Meier curves of follow-up in the health care claims data according to age

Figure 3 shows the age-specific Kaplan–Meier curves of the follow-up durations from each participant’s earliest recorded month to the latest recorded month within the health care claims data. According to age group, the 3-year follow-up rates were 46.2% for 0–19 years, 30.2% for 20–39 years, 53.5% for 40–59 years, 83.1% for 60–79 years, and 71.6% for ≥80 years; the 4-year follow-up rates were 32.2% for 0–19 years, 20.1% for 20–39 years, 42.9% for 40–59 years, 76.8% for 60–79 years, and 62.0% for ≥80 years.

## DISCUSSION

In 2019, we launched the LIFE Study as a multi-region community-based database project to generate evidence toward reaching Japan’s health policy targets of extending healthy life expectancy and reducing health disparities. Using heterogeneous multi-source data centered on health care claims data, the LIFE Study’s participants cover all ages from birth to death. In this way, the LIFE Study is facilitating new research in Japan through the provision of a real-world database comprising over 1 million participants that encompass a variety of diseases. By developing the LIFE CDM, we are able to integrate and standardize the stored data derived from multiple sources. The LIFE Study is expected to be utilized by diverse groups of researchers and contribute to the undertaking of data-driven research with real-world applications. While the 3-year follow-up rate for participants aged 20–39 years was relatively low at 30.2%, the follow-up rates for participants aged ≥60 years were high at over 70%. This enables medium-to-long term follow-up analyses using various study designs, such as cohort studies and nested case-control studies.^9^^–^^12^

In 2011, the National Database of Health Insurance Claims and Specific Health Checkups of Japan (NDB) began providing data for research. The NDB encompasses health care claims data and LTC claims data from almost all citizens and long-term residents within Japan, so it has the potential to support a wide variety of population-based research projects. However, there are certain advantages that the LIFE Study offers over the NDB. First, the NDB requires several months from application to data provision, so it cannot support the rapid generation of evidence for time-sensitive topics (eg, COVID-19). In contrast, the LIFE Study can facilitate research that should be released quickly when needed, such as a study on changes in percutaneous coronary intervention practice during the first wave of COVID-19 in Japan.^12^ As municipal governments can access health care claims data approximately 2 months after health services are provided, the LIFE Study is able to collect data with only a 2- or 3-month time lag. Second, the NDB stipulates that aggregate data with only 1 to 9 cases cannot be published, which can cause gaps in the evidence (eg, when mortality rates cannot be reported for certain groups).^13^ This is a potentially critical limitation for safety-related epidemiological studies, such as studies on pharmaceutical products and vaccines. In the LIFE Study, the participating municipal governments may check any results before publication but impose no restrictions on the minimum number of cases that can be reported in aggregate data. This allows the reporting of rare events as needed. Third, the NDB only provides researchers with the minimum numbers of patients and data items required for each research application. This can impede exploratory analyses or subgroup analyses, such as analyzing data from matched control patients in nested case-control studies. In addition, this approach is unsuitable for studies that use disease prediction models, which require a large variety of data items. In contrast, the LIFE Study allows the flexible provision of data in accordance with each researcher’s study design, thereby enabling the most effective use of its database. Fourth, studies on health disparities using the NDB have been limited to prefecture-level analyses,^14^^–^^16^ and little is known about the disparities according to socioeconomic status. While the NDB is scheduled to start providing information on patients’ areas of residence and income levels from April 2022, the provision of such data will be subject to even more stringent screening processes. This may hamper the use of these data even when they become available. The LIFE Study already collects data on residence-related information, out-of-pocket payment ceiling amounts, and insurance premium categories; and researchers may acquire these data through standard application and screening procedures.

The LIFE Study has been utilized in several cohort studies.^9^^–^^12^ There are numerous community-based cohort studies that have been or are being conducted in Japan. However, those studies generally have more narrow foci tailored to their research objectives, such as investigations on the primary endpoints of specific diseases, age groups, regions, and data items. For example, many cohort studies have focused on diseases, such as cancer,^13^^–^^20^ heart disease,^14^^,^^15^^,^^17^^–^^19^^,^^21^^–^^24^ stroke,^14^^,^^19^^,^^21^^–^^24^ and dementia.^23^ In contrast, some other cohort studies have examined multiple diseases.^15^^–^^18^^,^^23^^,^^25^^–^^31^ Among the cohort studies conducted on specific age groups, few have focused on children,^26^^,^^28^^,^^29^ whereas more have analyzed adults^13^^–^^24^^,^^32^ and older persons.^21^^–^^25^^,^^27^^,^^31^^,^^32^ We were unable to find any Japanese studies that covered all age groups within a single cohort. For region-specific research, several studies have focused on a single municipality,^19^^,^^23^^,^^25^^,^^30^^–^^32^ while others have analyzed multiple municipalities.^13^^–^^18^^,^^20^^–^^22^^,^^24^^,^^26^^–^^29^ With regard to data items, the majority of cohort studies have used questionnaire-based surveys,^13^^–^^15^^,^^19^^,^^20^^,^^22^^–^^32^ data collected specifically for research,^13^^–^^18^^,^^20^^,^^22^^–^^26^^,^^28^^,^^31^^,^^32^ and genetic information^13^^,^^15^^,^^23^^,^^26^^,^^28^; these data types are highly useful in that they provide detailed measurements. However, to the best of our knowledge, there are no Japanese cohort studies that have used multi-source data centered on health care claims data, which are available at a national level. When compared with the above studies, the LIFE Study is distinctive in its ability to support life course research on all diseases in all age groups. The LIFE Study currently receives data from 18 municipalities across four prefectures, and this number will continue to rise. Although the data items used by the LIFE Study may be less detailed that those of previous cohort studies, the incorporation of various health checkup and screening information may enable investigations of health problems that were previously not assessable. Furthermore, we launched a new project based on the LIFE Study in 2021 named the LIFE Sustaining Health by Integrating Next-generation Ecosystems (LIFE-SHINE) Study. This new project collects detailed data, such as salivary diagnostic test data (eg, cariogenic bacteria, acidity, and ammonia levels), pedometer measurements, and lifelog records during specific health checkups and LTC prevention classes conducted in participating municipalities. The LIFE-SHINE Study will provide detailed measurement data as a baseline for subsequent follow-up by the LIFE Study, thereby enabling large-scale prospective studies to be conducted at extremely low cost.

A strength of the LIFE Study that distinguishes it from existing databases and cohort studies is the secondary collection of multiple datasets (health care insurance data, LTC insurance data, health data, and government administration data) already stored by municipal governments, and their tertiary processing into an integrated database with research applications. This approach removes potential causes of biases frequently experienced by community-based cohort studies, such as low participation rates and low response rates. Also, as the data are already available in the municipalities, their collection does not entail additional costs. While the continuation of cohort studies is generally contingent on the acquisition of research funds, the LIFE Study is able to minimize the costs associated with data collection. Another strength of the LIFE Study is the construction of health platforms in the participating municipalities that can link various data types with health care claims data. In 2021, we introduced the LIFE Vaccine Effectiveness, Networking, and Universal Safety (LIFE-VENUS) Study, which links health care claims data and vaccination records. Previously, vaccine-related information was limited to data collection systems with single-arm designs, such as adverse event reports from vaccinated individuals. As a consequence, researchers were unable to verify the causal relationships in the safety and effectiveness of vaccines using local data. However, the LIFE-VENUS Study enables us to ascertain the vaccination statuses of individuals from vaccination records, and the health care claims data provide insight into the subsequent occurrence of health-related events. This allows the implementation of vaccine postmarketing surveillance within the participating municipalities. Similar approaches could also be used to assess the effectiveness of LTC prevention services, dental checkups, and cancer screenings. Finally, the LIFE Study allows comparisons of specific health indicators among different age groups, socioeconomic groups, and regions, thereby shedding light on potential disparities. By identifying these disparities and their underlying causes, corrective measures could be designed and implemented.

Despite the above strengths, the LIFE Study is not without limitations. First, non-negligible proportions of children and working-age adults are not enrolled in government-administered social insurance schemes (including pension and health insurance), so they are not included in the LIFE Study’s participants. In contrast, the vast majority of older persons in Japan are enrolled in either National Health Insurance or the Latter-Stage Older Persons Health Care System, and their high inclusion rates in the LIFE Study would increase their subpopulation’s representativeness. However, most health problems tend to appear more frequently in older persons, and there are commercially available claims datasets from private health insurance that do not cover older persons. In consideration of these points, the LIFE Study offers a new and useful data environment that can be applied to public health and health services research. Second, the LIFE Study lacks information on lifestyle factors and genetic determinants, which are important variables that feature prominently in recent large-scale community-based cohort studies. Although the LIFE Study does include some information on lifestyle factors obtained from medical questionnaire forms and social engagement levels from needs assessment forms, there are considerable limits to the number of respondents and data items included. The inclusion of further questionnaires on lifestyle factors during health checkups and needs assessments may help to overcome this limitation. The addition of various detailed measurement data from the LIFE-SHINE Study can also help to increase the breadth of data. Third, the LIFE Study is based on individual agreements with each municipality, which could affect its continuity over time. This has also resulted in variations in the coverage of participants and data items among the municipalities, depending on what each municipal government has decided to share. To ensure the continuity of the LIFE Study, we are working to provide informative and useful analytical results as feedback to the participating municipal governments, thereby building a mutually beneficial relationship between the municipalities and researchers.

### Conclusion

The LIFE Study was created as a large-scale multi-region community-based database project aimed at facilitating research to extend healthy life expectancy and reduce health disparities in Japan. While the LIFE Study currently has fewer detailed data items than previous community-based cohort studies, it provides a study population in excess of 1 million residents covering all age groups and diseases. The LIFE Study is expected to be a useful platform for generating real-world evidence from Japan.
